# Expanding the pH‐Shift Technique for Sequential Extraction of Intact Proteins and Sulfated Polysaccharide From Fish Heads: A Novel Biorefinery Approach

**DOI:** 10.1002/fsn3.70673

**Published:** 2025-07-30

**Authors:** Shahab Naghdi, Masoud Rezaei, Mehdi Tabarsa, Mehdi Abdollahi

**Affiliations:** ^1^ Seafood Processing Department, Marine Sciences Faculty Tarbiat Modares University Noor Iran; ^2^ Department of Biology and Biological Engineering–Food and Nutrition Science Chalmers University of Technology Gothenburg Sweden

**Keywords:** bioactive and functional properties, fish by‐products, pH shift, protein isolates, sequential extraction, sulfated polysaccharides

## Abstract

The pH‐shift technology was successfully expanded for the sequential recovery of proteins and sulfated polysaccharides (SPs) from rainbow trout heads. Adjusting the pH of the remaining process water after protein recovery at their isoelectric point to 8 enabled precipitation and recovery of SPs with the aid of ethanol at both alkaline and acid process versions and named SP‐11.5 and SP‐2.5, respectively. The mass yield of SPs recovered using the alkaline process version was 3.25%, nearly double that of SP (1.75%) from the acid version. SP‐11.5 contained higher levels of carbohydrates (61.22%), proteins (13.29%), and sulfates (12.13%) compared to SP‐2.5. FTIR, DSC, and XRD analyses showed no significant differences in the structural properties of the recovered SPs as a function of the pH‐shift process version. However, SP‐11.5 exhibited better antioxidant activity in DPPH, ABTS, and metal chelating tests and superior antibacterial properties against *Listeria monocytogenes* and *Escherichia coli* than SP‐2.5. This study suggests that the pH‐shift process can be effectively extended for sequential extraction of both protein isolates and SPs from fish by‐products for a multiple product biorefinery where the alkaline version outperformed.

## Graphical abstract

The pH‐shift technology was successfully expanded for the sequential recovery of proteins and sulfated polysaccharides (SPs) from rainbow trout heads. Adjusting the pH of the remaining process water after protein recovery at its isoelectric point to 8 enabled precipitation and recovery of SPs with the aid of ethanol at both alkaline and acid process versions.

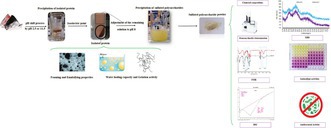

## 1 Introduction

Recently, changes in lifestyle and growing awareness of seafood's nutritional benefits have led to an increase in global seafood consumption. This shift is evident from global seafood production exceeding 223.2 million tons in 2024, with aquaculture contributing 130.9 million tons (FAO 2022). Rainbow trout ( *Oncorhynchus mykiss* ), a major aquaculture species, accounted for nearly 740,000 tons globally, with Iran producing over 212,000 tons (FAO 2022). Significant quantities of fish are transformed into byproducts such as heads, bones, and viscera, which are often undervalued despite being rich in proteins, essential amino acids, SPs, and omega‐3 fatty acids (Nikoo et al. 2021; Pezeshk et al. 2021; Zhang et al. 2024). These byproducts have increasingly been utilized to produce oil and fish meals, although they are rich in high‐quality protein, essential amino acids, sulfated polysaccharides (SPs), *n*−3 polyunsaturated fatty acids, and minerals (Baraiya et al. 2024; Sila et al. 2018; Zhang et al. 2024). The increasing importance of sustainable food production, coupled with growing consumer demand for healthy marine foods and bioactive compounds, necessitates the efficient use of fish byproducts (Baraiya et al. 2024; Nikoo et al. 2021).

SPs are diverse, large molecules, anionic polysaccharides found in a wide range of terrestrial organisms, including animals, fish, plants, and microorganisms that play a crucial role in cell communication and recognition (Hou et al. 2025; Nogueira et al. 2019; Paulose and Chakraborty 2025). Also, they are characterized by sulfate groups attached to their hydroxyl groups and have a relatively low molecular weight that exhibits a wide range of biological functions, including antimicrobial, antioxidant, and anticancer properties (Gaspar‐pintiliescu et al. 2024). These compounds are bound to proteins in different tissues, necessitating their separation through various extraction methods, including enzymatic hydrolysis (Jridi et al. 2018), ultrasound‐enzymatic hydrolysis (Naghdi et al. 2024, 2023), microwave‐enzymatic hydrolysis (Wang et al. 2011), hot pressure (Shen et al. 2019), and sodium acetate treatment (Nakano et al. 1998). However, since SPs are minor components in aquatic side streams, there is an intense economic and environmental motivation for their parallel or sequential extraction with other valuable compounds, especially proteins, as part of a biorefinery platform (Mkadem and Kaanane 2024; Tsegay et al. 2024). For instance, Shen et al. (2019) recently successfully expanded the hot‐pressure technology to a biorefinery process for the co‐production of chondroitin sulfate and peptide from liquefied chicken sternal cartilage.

Another alternative technology that has gained great interest for the extraction of intact gel‐forming proteins from aquatic side streams is the so‐called pH‐shift method. The pH‐shift process, involving acid and/or alkaline solubilization of proteins followed by their isoelectric precipitation, can handle complex raw materials very effectively and gently. In contrast to enzymatic hydrolysis, the pH‐shift technology separates proteins from other side‐stream components (e.g., bones and fats) without inducing protein degradation. Additionally, the quality of seafood proteins and lipids is better preserved since the entire pH‐shift process is conducted at cold temperatures. Furthermore, proteins recovered using the pH‐shift process can be used in wet form (e.g., as mince) for product development, which avoids the energy‐intensive drying step required at the end of the enzymatic hydrolysis process, thereby expanding the product's application potential. On top of that, the process has been successfully transformed into a biorefinery platform for the sequential extraction of gel‐forming proteins, collagen, collagen hydrolysate, and fish oil (Abdollahi et al. 2018; Chumthong et al. 2024; Mkadem and Kaanane 2024; Naghdi et al. 2025). However, an overlooked potential of the pH‐shift process is its ability to act as a simpler and more cost‐effective method for disrupting SP‐protein linkages under acidic or alkaline conditions, releasing SPs for subsequent recovery from the processing water. Nonetheless, this method might pose a higher risk of degrading SPs, potentially reducing their molecular weight and bioactivity, highlighting the need for detailed investigation to optimize conditions and maximize SP recovery, which remains a gap, to the best of our knowledge.

This study, therefore, presents the first evaluation of the potential of the pH shift method at both alkaline and acidic versions as a novel multiple‐product biorefinery approach for sequentially extracting proteins and SPs from rainbow trout heads. Furthermore, we assessed the effect of the extraction condition on the chemical composition, functional properties, and bioactivity of the extracted SPs.

## 2 Materials and Methods

### 2.1 Reagents and Chemicals

The reagents used in this work were obtained from Sigma branches: 1,1‐diphenyl‐2‐picrylhydrazyl (DPPH), potassium ferricyanide, ascorbic acid, ethylenediaminetetraacetic acid (EDTA), 2,2′‐azino‐bis (3‐ethylbenzothiazoline‐6‐sulphonic acid) (ABTS), monosaccharide standards, and trifluoroacetic acid (TFA). All other materials and solvents were of high purity and analytical grade.

### 2.2 Sample Collection

The head of a rainbow trout ( *O. mykiss* ) was provided by a local market (Nour, Iran). We had already coordinated with the local fishmonger to prepare the sample. After preparing the samples, we transferred them in the presence of crushed ice (the distance between the store and the university is about 5 min). Upon arriving at the fish processing laboratory, the samples were rinsed with tap water to eliminate any pigments, then packed in bags and stored at −18°C until they were used.

### 2.3 Protein Isolation Using the pH‐Shift Method

To isolate protein from the heads, ground samples were homogenized with cold distilled water (4°C) at a 1:4 (w/v) ratio using a high‐speed homogenizer (15,000 rpm for 2 min). The mixture's pH was then adjusted to 11.5 and 2.5 using 2 M NaOH and HCl, respectively. After stirring for 5 min to promote solubilization, we centrifuged the homogenates at 8500 × g for 20 min at 4°C. The upper lipid layer was removed, and the supernatant was collected for isoelectric precipitation. This supernatant is then adjusted to a pH of 5.5 using 2 M HCl and NaOH and maintained at this pH with constant stirring and cooling in an ice bath for 10 min. Then, the second centrifugation step was done by centrifuging the protein slurry at 4°C for 20 min at 8500 × g. Next, the pH of the precipitated protein was adjusted to 7 using 2 N NaOH in the presence of an ice bath. Finally, freeze samples were lyophilized using a freeze‐drier (FD‐5003‐BT, Mall Kala, Iran) at −85°C for 4 days. The obtained isolated protein from pH 11.5 and 2.5 was named IP‐11.5 and IP‐2.5, respectively, and stored at −80°C until their use.

### 2.4 Recovery of SPs From the Remaining Liquids of the Protein Isolation Step

**FIGURE 1:**
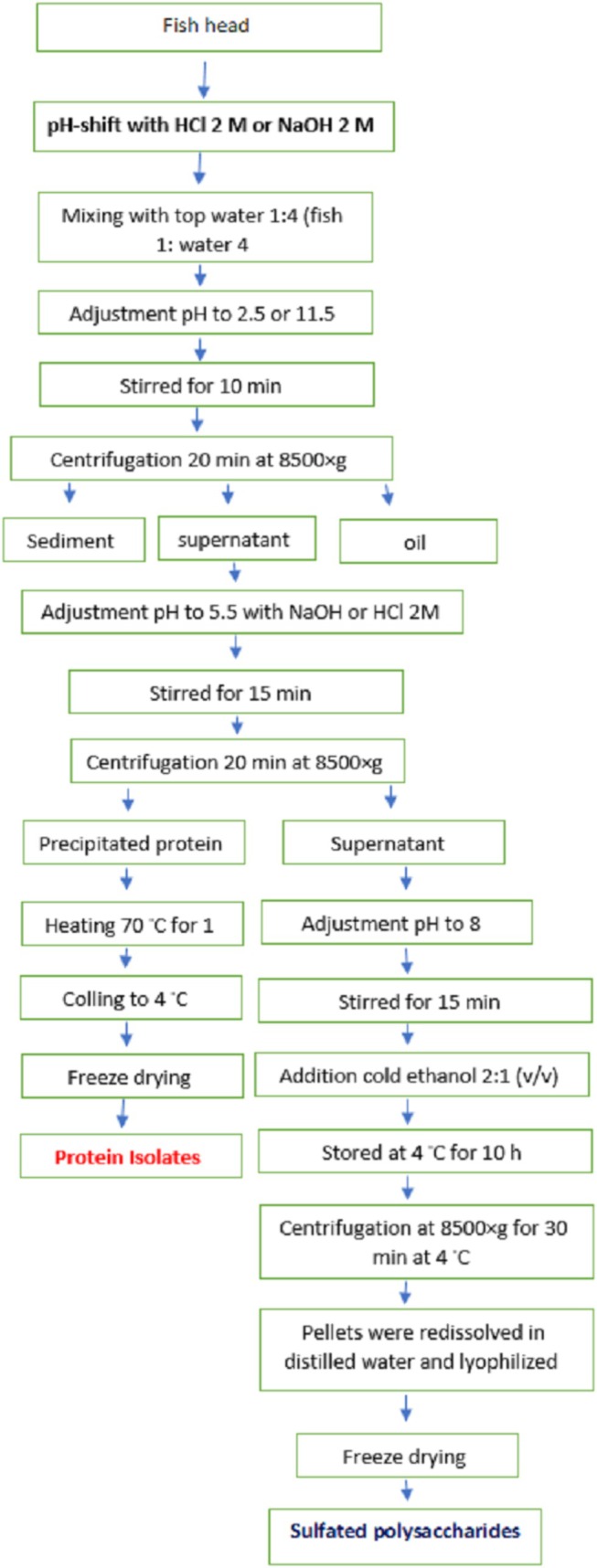
Schematic overview of the developed process for sequential extraction of proteins and sulfated polysaccharides using the pH shift method.

After isolating the fish head protein at pH 5.5, the remaining liquid was used to recover SPs (see Figure 1). Since the pH of the remaining extracts was around 5.5, we employed two procedures for the extraction of SPs:

First, we adjusted the pH of the remaining liquid to 7.5 (neutral) and then precipitated the sulfated polysaccharides using cold ethanol at a ratio of 2:1 (2 parts ethanol to 1 part liquid).

Second, the remaining liquid from the protein isolation step without changing the pH was used to isolate the SPs. The extracts were then stored at 4°C for 12 h. Subsequently, they were centrifuged at 9000 rpm for 20 min at 4°C. After this step, it was determined that SPs only precipitated in the method where the pH of the remaining liquid was adjusted to 7.5. The reason for the present results could be because at pH 8, some acidic groups on sulfated polysaccharides become partially deprotonated, which reduces the net positive charge and slightly decreases their solubility in water. Additionally, the addition of cold ethanol lowers the dielectric constant of the medium, disrupting the hydration shell and further reducing solubility, leading to the aggregation and precipitation of the sulfated polysaccharides (Chen et al. 2023; Hu and Goff 2018). Finally, the precipitated SPs were suspended in distilled water and lyophilized. The obtained samples were stored in the freezer until further use. Different SPs obtained from the fish head by acid (2.5) and alkaline pH (11.5) solubilization were named SP‐2.5 and SP‐11.5, respectively. The process of deproteinization of the samples was carried out using the Sevage method, as described in the protocol by Yang et al. (2023), before precipitation with ethanol.

### 2.5 Measurement of Protein Yield and Solubility of the IPs

Protein yield and solubility were determined for both acid and alkaline processing methods using the Lowry method (Lowry, Rosebrough, Farr, and Randall 1951). This method measured the protein content of the samples. In brief, 1 mL of appropriately diluted protein sample (0.1–1.0 mg/mL) was mixed with 5 mL of alkaline copper sulfate reagent (containing 2% Na_2_CO_3_, 1% CuSO_4_·5H_2_O, and 2% potassium sodium tartrate in 0.1 N NaOH), and incubated at room temperature for 10 min. Then, 0.5 mL of diluted Folin–Ciocalteu reagent (1:1 with water) was added. The mixture was incubated in the dark for 30 min, and absorbance was measured at 660 nm. Protein concentrations were determined using a standard curve prepared with bovine serum albumin (BSA).

### 2.6 Determination of Free Sulfhydryl Groups (SH) of the IPs

The concentration of free sulfhydryl (–SH) groups in the protein isolates was determined using Ellman's reagent (5,5′‐dithiobis (2‐nitrobenzoic acid), DTNB), with slight modifications based on the method described by He et al. (2014). Briefly, both the protein isolate solution (prepared at 3 mg/mL) and the DTNB reagent were separately dissolved in tris–glycine buffer. The protein solution was then centrifuged at 2500 × g for 10 min, and the resulting supernatant was collected. Its protein concentration was determined using the same procedure described in Section 2.5. Subsequently, 50 μL of the DTNB solution was added to 5 mL of the protein supernatant. The reaction mixture was allowed to incubate at room temperature for 30 min. The absorbance was then measured at 412 nm using a UV–VIS spectrophotometer. A sample without DTNB served as the blank. The sulfhydryl content was calculated using the following formula:

(1) $$\mathrm{SH}\;\left(\text{μmol}/g\right)=73.53\times A\times D/C$$

where A is the absorbance at 412 nm, D is the dilution factor, and C is the protein concentration of the supernatant (mg/mL) (Ellman 1959).

### 2.7 Tryptophan Intrinsic Fluorescence Intensity of the IPs

The intrinsic fluorescence intensity of tryptophan in the IP samples was measured using a fluorescence spectrophotometer (F‐4500, Hitachi, Japan). Protein samples were prepared at a concentration of 0.15 mg/mL. The excited samples by wavelength of 280 nm were used to record fluorescence emission at 325 nm (Chanthai et al. 1996). Each sample will be scanned three times to ensure accuracy.

### 2.8 Evaluation of Functional Properties of IPs

#### 2.8.1 Emulsifying Properties of IPs

The emulsifying features including activity index (EAI) and emulsifying stability index (ESI) of the isolated proteins were measured according to the method described by Xi et al. (2020) with slight modifications. For this purpose, 3 mL of corn oil was homogenized with 5 mL of a 1% solution of the fish protein sample using a laboratory homogenizer at 15,000 rpm for 2 min at room temperature. Immediately after homogenization and 10 min later, 50 μL of the emulsion from the bottom of the container was taken and homogenized in 5 mL of a 0.1% sodium dodecyl sulfate (SDS) solution. This mixture was then vortexed for 10 s. The absorbance of the solution was then measured using a spectrophotometer at a wavelength of 500 nm, and the EAI and ESI were calculated using the following equations:

(2) $$\mathrm{EA}\;\left(m^{2}/g\right)=\left(2\times 2.303\right)/\left(C\times \left(1-\varphi \right)\times 10\right)\times \text{A0}\times \text{dilution}$$

(3) $$\mathrm{ESI}\;\left(\%\right)=A10/\text{A0}\times 100$$

where C is the initial protein concentration (mg/mL), φ represents the oil volume fraction (v/v) of the emulsion (0.25), 100 is the dilution factor, and A0 and A10 are the absorbances of the diluted emulsion at 0 and 10 min, respectively.

#### 2.8.2 Foaming Properties of IPs

Foam capacity (FC) and foam stability (FS) of the IP samples were assessed using the method outlined by (Elsohaimy et al. 2015). The protein samples (100 mg) were homogenized in 10 mL of distilled water with a laboratory homogenizer at 15,000 rpm for 2 min at room temperature. Foam capacity and stability were then calculated using the following formulas:

Foam capacity (FC) was determined by comparing the foam volume immediately after homogenization (V1) to the initial liquid volume of the samples (10 mL). Foam stability (FS) was measured by comparing the foam volume at 30 s (V2) to the initial foam volume (V1).

(4) $$\mathrm{FC}\;\left(\%\right)=V1/10\times 100$$

(5) $$\mathrm{FS}\;\left(\%\right)=V2/\text{V1}\times 100$$

#### 2.8.3 The Minimum Gelation Concentration of IPs

The minimum gelation concentration of the IP samples was evaluated using the method reported by Benelhadj et al. (2016). The protein concentrations of 3%, 6%, 9%, 12%, and 15% were prepared in distilled water and then incubated in a water bath at 100°C for 1 h. Subsequently, the samples were rapidly cooled using running water and then stored at 4°C for 2 h. The lowest concentration at which the solution did not fall when the test tube was inverted was considered the minimum gelation concentration.

### 2.9 Chemical and Monosaccharide Compositions of Recovered SPs

#### 2.9.1 Chemical Composition of SPs

The carbohydrate content in the recovered SPs was determined using the Dubois method (Dubois et al. 1956). In brief, 1 mL of the sample protein (SP) solution was combined with 1 mL of 5% phenol, followed by the addition of 5 mL of concentrated sulfuric acid. The mixture was vortexed thoroughly and then left to stand at room temperature for 30 min to allow color development. The absorbance was recorded at 490 nm using a UV–VIS spectrophotometer. Quantification was performed against a glucose standard curve. The amount of protein in the SP samples was assessed using the Lowry method (Lowry, Rosebrough, Farr, Randall, Lowry, et al. 1951). A conventional method utilizing barium chloride and gelatin was employed to estimate the sulfate content of the SPs (Loyd et al. 1960). Briefly, SP samples underwent acid hydrolysis to liberate sulfate ions. A portion of the hydrolysate was combined with gelatin solution, then barium chloride was added to form a barium sulfate precipitate. The turbidity caused by this precipitate was measured at 360 nm using a spectrophotometer. Sulfate concentrations were determined by referencing a potassium sulfate standard curve. The total uronic acid content was quantified using a colorimetric method published by (Bitter and Muir 1962). SP samples were treated with sulfuric acid and carbazole reagent under controlled heating to develop a red–purple chromophore. Following cooling, the absorbance was measured at 530 nm. Quantification was performed using a galacturonic acid standard curve.

#### 2.9.2 Determination of Monosaccharide Composition of SPs

According to Naghdi et al. (2023) a GC–MS system was employed to analyze the monosaccharide composition of the recovered sulfated polysaccharides (SPs). This system featured an HP‐5 column measuring 30 m in length, with an internal diameter of 0.32 mm. The temperature protocol for the instrument began at an initial temperature of 120°C, which was gradually increased to 240°C at a rate of 10°C per minute, followed by a 6‐min hold at this maximum temperature. Both the detector and injector were set to 250°C. Nitrogen served as the inert carrier gas, and standards used for the analysis included rhamnose, xylose, mannose, glucose, and galactose.

### 2.10 Structural Characterization of SPs

#### 2.10.1 Fourier Transform Infrared (FTIR) Spectroscopy Analysis of SPs

To specify the functional group of SPs, they were mixed with KBr and placed into the testing pellet. Then, their FTIR spectrum was recorded using an FTIR spectrophotometer at room temperature, covering a range from 4000 to 400 cm^−1^ with a resolution of 4 cm^−1^ (Naghdi et al. 2023).

#### 2.10.2 Differential Scanning Calorimetry (DSC) and Thermogravimetric Analysis (TGA) of SPs

Five milligrams of the SP samples were weighed and placed in an aluminum pan, and their moisture content was adjusted to 70% by adding distilled water. The samples were then sealed and left at room temperature for 1 h. Following this, the samples were placed in a calorimeter and heated from 25°C to 200°C at a rate of 5°C per minute (Trigui et al. 2018).

#### 2.10.3 X‐Ray Diffraction of SPs

To analyze the crystallinity patterns of the extracted SPs, XRD analysis was conducted using a Siemens D5000 X‐ray diffractometer (Germany) equipped with a CuKα radiation source (λ = 1.54056 Å), operating at 40 kV and 30 mA. The samples were scanned over a 2θ range of 0°–80° at a rate of 0.5°/s at room temperature (Getachew et al. 2019).

### 2.11 Determination of Antioxidant Activities of SPs

#### 2.11.1 ABTS Scavenging Activity of SPs

The ABTS free radical scavenging activity of the recovered SPs was investigated using the method described by Naghdi et al. (2023). Firstly, a 7 mM solution of ABTS stable radical was prepared in distilled water. Then, a 2.45 mM potassium persulfate solution was added, and the mixture was kept in the dark for 16 h to form blue‐green cations. The final solution was then diluted until it reached an absorbance of 0.7 at 734 nm. Next, 0.5 mL of various concentrations of the samples (1, 2, and 3 mg/mL) was added to 1.5 mL of the prepared 0.7 mM ABTS solution. A control reaction was run using a mixture of ABTS and ethanol. The resulting solution was incubated in a microplate and read at 734 nm. The ABTS free radical scavenging activity was then calculated using the following formula:

(6) $$\text{ABTS scavenging activity}\;\left(\%\right)=\left(\mathrm{Ac}–\mathrm{As}\right)/\mathrm{Ac}\times 100$$

where Ac is the absorbance of the control sample (0.5 mL ethanol with 1.5 mL ABTS solution) and As is the absorbance of the sample.

#### 2.11.2 Iron (Fe^2+^) Chelating Activity of SPs

The ferric‐reducing power of SP samples was determined using the method described by Naghdi et al. (2023). For this purpose, 2.5 mL of 0.1 M potassium phosphate buffer (pH 6.6) and 2.5 mL of 1% potassium ferricyanide were mixed with 1 mL/g of the samples. This solution was incubated at 50°C for 20 min, after which 2.5 mL of 10% trichloroacetic acid was added, and the mixture was then centrifuged at 3000 rpm for 10 min. Subsequently, 2.5 mL of water and 0.5 mL of 0.1% FeCl_3_ were added to 2.5 mL of the prepared mixture. This solution was then incubated at a constant temperature for 30 min. After this time, the absorbance of the samples was read at 700 nm.

(7) $$\text{Metal chelating activity}\;\left(\%\right)=\left[\left(\mathrm{ODC}+\mathrm{ODB}-\mathrm{ODS}\right)/\mathrm{ODC}\right]\times 100$$

where ODC, ODB, and ODS describe the control, the blank absorbance (positive control was readied like other samples but substituting SPs with distilled water) and the sample reaction tubes, respectively.

#### 2.11.3 DPPH Scavenging Activity of SPs

The antioxidant activity of the extracted SPs was investigated using the stable free radical 2,2‐diphenyl‐1‐picrylhydrazyl (DPPH) (Naghdi et al. 2023). In this experiment, 2 mL/g of the samples were added to 2 mL of 0.16 mM methanol solution of DPPH free radical and shaken for 1 min. The mixture was then incubated at room temperature for 30 min, and the absorbance of the solution was read at a wavelength of 517 nm. The DPPH radical scavenging activity of the samples was calculated using the following formula:

(8) $$\mathrm{RSA}=\left[1-\left(A_{\text{sample}}–A_{\text{blank}}\right)/A_{\text{control}}\right]\times 100$$

A_sample_ = Absorbance of the sample after the specified time (sample and solution containing DPPH).

A_control_ = Absorbance of the solution containing free radicals without the sample.

A_blank_ = Absorbance of the sample after the specified time (sample without the solution containing DPPH).

### 2.12 Antibacterial Activity of SPs

#### 2.12.1 Bacterial Strains

*Listeria monocytogenes* (CMCC 54,007) and *Escherichia coli* (O157:H7) were used as the two most important food‐borne human pathogens, obtained from the Pasteur Institute of Iran.

#### 2.12.2 Agar Diffusion Method

A density of 1 × 10^5^ CFU/mL of the two bacterial strains mentioned was used to evaluate the antibacterial activity of SPs by uniformly spreading them on the Tryptic Soy Agar medium surface. Sterilized punched discs were then soaked in 50 μL of the concentrations (1 mg/mL) of SP samples. Next, the discs were placed on the plate surfaces. After incubation of the plates at 37°C for 24 h, the diameter of the inhibition zone was recorded in millimeters. These experiments were conducted in triplicate.

### 2.13 Statistical Analysis

Statistical analyses were carried out by SPSS ver. 22.0; one‐way analysis of variance (ANOVA) and Duncan's multiple range test were used to evaluate the significant differences between the variables. The experimentations were done in triplicate. Differences were considered significant at *p* < 0.05.

## 3 Results and Discussion

### 3.1 Protein Solubilization and Protein Precipitation Yield of the Produced IPs

Table 1 shows that the protein yield under acidic conditions (2.5) was 46.54%, whereas the alkaline condition (11.5) achieved 51.84%. Both samples exhibited solubility greater than 50%. These findings were consistent with previous studies conducted on various species like salmon, cod, herring, and rainbow trout (Pezeshk et al. 2021). The reason for this phenomenon can be attributed to the enhanced interaction between water and proteins in alkaline environments (Surasani 2018). This enhancement leads to improved protein solubility, ultimately resulting in a higher protein yield during the alkaline extraction process (Surasani 2018). The lower solubility of proteins in acidic processes is likely due to protein denaturation caused by acid; this denaturation is more severe in acidic processes than in alkaline pH shift processes, resulting in protein precipitation (Panpipat and Chaijan 2017). However, the content of proteins in the raw material along with their amino acid composition and structural characteristics also significantly influences protein yield and solubility at a specific pH level (Surasani 2018; Tortolero et al. 2016). Furthermore, the pretreatments applied to the raw material can lead to variations and differences in solubility between different species (Batista et al. 2007). This variability could explain the differences observed in our study compared to earlier research, even those involving the same species.

**TABLE 1:** Protein solubility, yield, and intrinsic fluorescence spectrum of protein isolated from *O. mykiss* using acid (IP‐2.5) and alkaline (IP‐11.5) versions of the pH‐shift method.

Samples	Yield (%)	Solubility (%)	Intrinsic fluorescence spectrum	Reactive SH content (μmol/g)
IP‐2.5	46.54 ± 1.07^b^	53.90 ± 1.06^b^	2442.67 ± 85.80^b^	17.23 ± 1.05^a^
IP‐11.5	51.84 ± 0.62^a^	61.32 ± 1.44^a^	3310.33 ± 65.57^a^	8.48 ± 0.99^b^

*Note:* The results are expressed as mean ± standard deviation. Different superscript letters indicate significant differences between means.

### 3.2 Tryptophan Fluorescence Intensity of the IPs

Protein fluorescence derived from tryptophan residues can serve as a valuable signal of modifications in protein conformation (Cai et al. 2019; Jiang et al. 2009). We measured the intrinsic fluorescence of tryptophan to assess protein conformational changes and the results are presented in Table 1. IP‐11.5 showed significantly greater fluorescence than IP‐2.5, suggesting reduced structural unfolding under alkaline conditions. This result could be related to more significant structural alterations in acidic conditions or the possibility of protein structure reverting more easily under alkaline treatments (Xu et al. 2012). Similarly, Nolsøe and Undeland (2009) detected reduced tryptophan fluorescence intensity in protein isolates produced in acidic conditions for kilka ( *Clupeonella cultriventris* ) fish compared to those obtained in alkaline conditions. They noted that as a protein's structure unfolds, amino acids present in the protein's internal hydrophobic space become exposed to the external hydrophilic and polar environment. Also, as the majority of intrinsic protein fluorescence stems from tryptophan, this exposure results in a decrease in tryptophan fluorescence intensity. Xu et al. (2012) showed that lowering the pH and acidifying the condition decreased the tryptophan fluorescence intensity of fish actomyosin, suggesting a greater unfolding of the natural protein structure and exposure of buried tryptophan groups to a more polar environment during the pH reduction process.

### 3.3 Reactive Sulfhydryl (R–SH) Group Content of the IPs

The availability of free sulfhydryl groups within protein structure can play a crucial role in determining the extent of disulfide bond formation during gel formation (Hu et al. 2023). As shown in Table 1, the concentration of free sulfhydryl groups in IP‐2.5 (17.23 μmol/g) was significantly higher than that of IP‐11.5 (8.48 μmol/g), and this difference was statistically significant (*p* < 0.05). Similar findings were reported in the studies by Jiang et al. (2009), who suggested that the lower concentration of R‐SH in alkaline pH conditions may be related to disulfide bond formation in proteins through SH/S‐S exchange reactions. Additionally, in alkaline pH conditions, thiolate groups are more likely to react with mercaptide ions, accelerating their oxidation. In a related study, Chaijan et al. (2006) reported higher amounts of active sulfhydryl groups in alkaline isolates of short mackerel compared to those obtained from acidic washes. Furthermore, they proposed that alkaline pH conditions can change protein charges, causing internal repulsion and consequently leading to conformational changes in the protein. It has also been reported that this reduction could potentially indicate the oxidation of sulfhydryl groups or SH/S‐S exchange reactions during solubilization in acidic pHs (Yongsawatdigul and Park 2004).

### 3.4 Functional Properties of Isolated Proteins

#### 3.4.1 Foaming Capacity and Stability of the IPs

Foams are described as two‐phase colloidal systems consisting of a continuous liquid phase and a dispersed air phase (Chalamaiah et al. 2015). Foaming activities are characterized by the transfer, permeation, and rearrangement of molecules at the air‐water interface, where adsorption at the air‐water interface is influenced by the hydrophobic regions in the molecules. Additionally, the amount of protein–protein interaction in the matrix impacts the film formation at the interface and determines the stability of the foam (Elavarasan et al. 2014). The foaming properties of the IP‐11.5 isolated protein sample were 77.00%, demonstrating significantly greater properties compared to the IP‐2.5 sample (*p* < 0.05) (Table 2). The results of assessing the stability properties of the foam samples mirrored the foaming results (*p* < 0.05). These results were aligned with the results reported by Pezeshk et al. (2021) and the possible explanation is the aggregation of recovered proteins at acidic pH, which restricts the ability of proteins to diffuse at the water/air interface to form bubbles. However, in the study of Panpipat and Chaijan (2017), the opposite results were reported to the present results, where the isolated protein obtained in alkaline and acidic pH from *Priacanthus tayenus* fish was 70% and 72%, respectively. They also stated that pH adjustment during dissolution and precipitation may significantly affect the foaming ability of protein isolates (Panpipat and Chaijan 2017). It has also been reported that the good foaming properties of isolated proteins can be related to flexible protein molecules that can reduce surface tension. In contrast, regular globular proteins, which are more resistant to denaturation, have less foaming ability and tend to exhibit lower foaming abilities (Graham and Phillips 1976). Hence, it may be suggested that acidic or alkaline pHs may cause structural changes in myofibrillar proteins (the primary proteins in protein isolates), exposing their internal hydrophobic amino acids to the inner surface of the air layer; this can enhance water absorption and induce them to behave like flexible proteins (Graham and Phillips 1976). On the other hand, it is well established that the foaming properties of proteins are mainly related to their solubility, the balance between flexibility and rigidity of proteins at the air‐water interface, hydrophobicity, pH, ionic strength, temperature, etc. (Elsohaimy et al. 2015). Among these factors, solubility stands out as the main factor influencing foaming properties, with higher solubility generally resulting in an increased foaming rate. The presented results related to the solubility of the samples (Table 1) revealed that the alkaline IP‐11.5 isolated protein exhibited greater solubility than that obtained from the acidic pH IP‐2.5.

**TABLE 2:** Functional properties of protein isolated samples from *O. mykiss* using acid (IP‐2.5) and alkaline (IP‐11.5) versions of the pH‐shift method.

	IP‐2.5	IP‐11.5
Foaming capacity (%)	55.00 ± 0.82^b^	77.00 ± 1.41^a^
Foaming stability (%)	39.39 ± 0.46^b^	63.64 ± 0.58^a^
Emulsifying activity index (EAI) (m^2^/g)	55.75 ± 0.96^b^	61.51 ± 2.92^a^
Emulsifying stability index (ESI) (%)	49.53 ± 1.52^b^	59.01 ± 1.32^a^
Water holding capacity (%)	25.87 ± 0.90^a^	27.90 ± 1.02^a^
Least gelation concentration (LGC) (w/w)	12%^a^	12%^a^

*Note:* The results are expressed as mean ± standard deviation. Different superscript letters indicate significant differences between means (*p* < 0/05).

#### 3.4.2 Emulsifying Properties and Stability of IPs

The ability of proteins to form emulsions is crucial in many food systems due to the interactions between proteins and lipids (Foh et al. 2012). The results of the emulsifying properties and stability of IPs are presented in Table 2. This evaluation revealed that the IP‐11.5 represented significantly higher emulsifying properties and emulsion stability than the IP‐2.5 sample (*p* < 0.05). Previous studies have shown that the emulsifying activity of the isolated proteins is influenced by their pH and molecular weight. For instance, Foh et al. (2012) reported that protein isolates obtained under alkaline conditions displayed better emulsifying properties than those obtained under acidic conditions, which was similar to our findings. These results can be attributed to the enhanced emulsifying capacity (EC) of protein isolates in alkaline conditions. The EC of proteins is influenced by the balance between their hydrophilic and lipophilic properties, which are affected by pH (Sathe et al. 1982). Studies have indicated that protein isolates obtained through pH shift processes possess a good ability to form a surface membrane during emulsifying tests. This enhances the dispersion of oil droplets compared to intact proteins (without pH shift processes). This is attributed to the increased exposure of hydrophobic amino acid side chains resulting from the partial unfolding of the protein structure in isolated samples, leading to increased surface activity (Jiang et al. 2009). Pezeshk et al. (2021) documented similar results, noting that fish protein isolates obtained at alkaline pH displayed higher emulsifying activity and emulsion stability than those obtained at acidic pH. They suggested that this might be due to the presence of highly negatively charged proteins in the alkaline isolates compared to the acidic isolates, enabling the proteins to spread more uniformly and effectively at the oil–water interface. Furthermore, in another study, it was noted that molecular flexibility and limited protein aggregation at the interface are crucial factors influencing the stability of oil‐in‐water emulsions (Jiang et al. 2009).

#### 3.4.3 Least Gelation Concentration

The results presented in Table 2 indicate that both isolated protein samples formed gels at a concentration of 12 g/100 g. Notably, before this concentration, none of the tested levels of the samples produced a stable gel that would remain intact when the Falcon tube was inverted. Interestingly, a study by Pezeshk et al. (2021) investigated the functional properties of rainbow trout protein isolate under various acidic and alkaline conditions. They found that isolated proteins produced at pH levels of 2.5, 3.5, and 11 had a minimum gel‐forming concentration of 10%. For pH 11.5, the minimum gel‐forming concentration was 20%. In contrast, the present study determined a minimum gel‐forming concentration of 12% for both samples. These findings align with those reported by Benelhadj et al. (2016), who also identified 12% as the minimum gel‐forming concentration for all their protein isolate samples. The gelation properties of protein samples are often related to partial protein denaturation, which allows functional groups within the protein to become exposed and interact with each other (Sathivel and Bechtel 2006). Additionally, strong hydrophobic interactions, as well as intermolecular hydrogen bonds and disulfide bonds, affect the thermal gelation behavior of proteins. Furthermore, differences in gel‐forming ability can be due to the variations in protein integrity and the bonds formed during heat treatment (Chaijan et al. 2006).

### 3.5 Yield and Chemical Component Compositions of Recovered SPs

Studies have demonstrated that marine animal polysaccharides are present in the skin, cartilage, secretory glands, muscles, and viscera of aquatic animals (Li et al. 2016). They exist as free polysaccharides or glycoproteins, some of which are linked to proteins via glycopeptide bonds. Polysaccharides are also found in various cellular components, including the cell membrane and intercellular and intracellular substances. Due to their unique properties in aquatic animals, two primary challenges arise during their extraction process. The first challenge is the rapid separation, migration, and dissolution of these polysaccharides, and the second challenge involves hydrolyzing the initial sources and their separation from their attached proteins while minimizing polysaccharide degradation (Xiong et al. 2018). As a result, different extraction procedures not only exhibit differences in extraction rate, yield, and purity but also directly impact the features, structures, and biological functions of polysaccharides (Balbinot‐Alfaro et al. 2021; Xiong et al. 2018). Table 3 presents the results of the yield and chemical composition evaluation of the obtained sulfated polysaccharides from rainbow trout heads using the pH‐shift method. The extraction yield of glycosaminoglycans (GAGs) recovered from the alkaline treatment (SP‐11.5) was 3.25%, which is approximately double that obtained from the acid treatment (SP‐2.5) (*p* < 0.05). This significant increase in yield under alkaline conditions can be primarily attributed to the enhanced solubilization of sulfated polysaccharides (SPs) facilitated by alkaline pH, which disrupts glycoprotein linkages more effectively than acidic conditions. Specifically, alkaline extraction promotes the cleavage of glycopeptidic bonds and the formation of soluble salts with acidic polysaccharides, thereby increasing their solubility and extraction efficiency (Xiong et al. 2018). Our findings are consistent with those of Naghdi et al. (2023), who reported similar extraction yields of SPs from skipjack tuna byproducts using enzymatic and ultrasound‐assisted enzymatic methods, underscoring the robustness of our approach. However, our extraction yield was lower than the results reported by Jridi et al. (2018), who achieved yields of 6.1% and 6.8% from squid skin and muscle, respectively. This discrepancy may be due to differences in raw material composition, extraction protocols, and the intrinsic variability of SP content among species and tissues, as previously noted by Abdelhedi et al. (2016) and Souissi et al. (2019). Moreover, the extraction method itself influences yield and composition; for example, enzymatic hydrolysis can lead to higher yields but may also cause partial degradation of polysaccharides (Wang et al. 2019). The alkaline extraction mechanism involves beta‐elimination reactions that break glycopeptide chains, releasing polysaccharides from protein complexes, as demonstrated in abalone gonad powder extraction (Song et al. 2018). However, it is crucial to control alkaline concentration because excessive alkalinity can degrade glycosidic bonds, leading to polysaccharide depolymerization and lower yields, as observed by Huang et al. (2009) and Li et al. (2016). Additionally, alkaline treatment may induce desulfurization of polysaccharides through the Walden inversion process, potentially affecting their sulfate content and bioactivity (Xiong et al. 2018). Supporting these observations, Bai et al. (2020) showed that increasing the extraction pH from acidic to neutral and alkaline conditions significantly enhanced polysaccharide yield, attributing this to improved solubility of acidic polysaccharides such as those from *Abelmoschus esculentus* L. Conversely, Wang et al. (2015) reported that at very high alkaline pH values (12–13), polysaccharide yields decreased due to degradation into smaller molecules that precipitate less efficiently during ethanol precipitation. Regarding the chemical composition, SP‐11.5 exhibited higher carbohydrate (61.22%), protein (13.29%), and sulfate (12.13%) contents compared to SP‐2.5, although only the carbohydrate content difference was statistically significant (*p* < 0.05) (Table 3). Interestingly, SP‐2.5 contained significantly higher uronic acid content (8.83%) than SP‐11.5, which may reflect differences in polysaccharide structure and extraction selectivity under acidic conditions. These compositional variations can be explained by the extent of protein and glycosaminoglycan hydrolysis during extraction, which affects molecular weight and structural integrity (Bai et al. 2020; Ye et al. 2019). Protein solubility is also pH‐dependent; at higher alkaline pH, proteins acquire additional negative charges, increasing electrostatic repulsion and solubility of low‐mobility proteins, which may explain the elevated protein content in SP‐11.5 (Ye et al. 2019). This interplay between protein dissolution and polysaccharide extraction highlights the complexity of the pH‐shift process and its impact on the final chemical composition of recovered SPs. Overall, these findings demonstrate that alkaline pH‐shift extraction not only improves SP yield but also influences their chemical characteristics, which may have implications for their functional and bioactive properties in downstream applications.

**TABLE 3:** Chemical and monosaccharide compositions, and extraction yield of sulfated polysaccharides (SP) recovered from *O. mykiss* by‐products using the acid (SP‐2.5) and alkaline (SP‐11.5) versions of the pH shift method.

	SP‐2.5	SP‐11.5
Chemical compositions (%)		
Total sugars	57.11 ± 2.11^b^	61.22 ± 2.17^a^
Total proteins	12.88 ± 1.13^a^	13.29 ± 1.41^a^
Uronic acid	8.83 ± 0.18^a^	6.70 ± 0.15^b^
Sulfated groups	11.45 ± 0.64^a^	12.13 ± 0.07^a^
Extraction yields (%)	1.75 ± 0.14^b^	3.25 ± 0.18^a^
Monosaccharide (%)		
Rhamnose	2.61 ± 0.66^b^	17.95 ± 0.19^a^
Mannose	11.02 ± 0.02^b^	17.14 ± 0.16^a^
Arabinose	ND	ND
Glucose	8.38 ± 0.17^a^	3.97 ± 0.12^b^
Galactose	77.96 ± 0.84^a^	60.91 ± 0.08^b^

*Note:* Data are calculated based on dry weights. Different superscript letters in the same row indicate significant differences (*p* < 0.05).

Abbreviation: ND, no detect.

The monosaccharide profiles of the extracted sulfated polysaccharide (SP) samples are presented in Table 3. Both SP‐11.5 and SP‐2.5 consisted primarily of rhamnose, mannose, glucose, and galactose, but their relative abundances differed significantly (*p* < 0.05). Notably, the SP‐11.5 sample obtained via alkaline extraction contained markedly higher levels of rhamnose (17.95%) and mannose (17.14%) compared to SP‐2.5, which contained only 2.61% rhamnose and 12.04% mannose. In contrast, SP‐2.5, recovered under acidic conditions, exhibited a significantly higher content of glucose (8.38%) and especially galactose (77.96%) than SP‐11.5 (*p* < 0.05). Interestingly, arabinose was not detected in any of the samples, indicating its negligible presence in the SPs extracted from rainbow trout heads under the tested conditions. The observed differences in monosaccharide composition between the two extraction methods can be attributed to the influence of pH on the solubilization and stability of specific polysaccharide fractions. Alkaline extraction conditions are known to promote the release of rhamnose‐ and mannose‐rich polysaccharide structures, likely due to the enhanced cleavage of glycoprotein linkages and increased solubility of certain acidic polysaccharides at higher pH (Xiong et al. 2018; Bai et al. 2020). Conversely, acidic conditions may favor the extraction or preservation of galactose‐rich polysaccharides, possibly due to selective precipitation or the stability of these structures at low pH. It is well established that both the source material and the extraction protocol play critical roles in determining the monosaccharide profile of recovered SPs. The intrinsic composition of the raw material, such as the tissue type and species, sets the baseline for the types and proportions of monosaccharides present (Abdelhedi et al. 2016; Jridi, Nasri, et al. 2019). Additionally, the extraction and purification procedures—such as pH adjustment, temperature, and the use of physical or enzymatic pre‐treatments—can selectively solubilize or degrade certain polysaccharide fractions, thereby altering the overall monosaccharide composition. For example, Chen et al. (2014) demonstrated that ultrasonic pretreatment during polysaccharide extraction could significantly modify the molecular ratios of monosaccharides, highlighting the sensitivity of SP composition to processing conditions. The high galactose content observed in SP‐2.5 aligns with previous findings in marine organisms; for instance, Zhu et al. (2011) reported similarly galactose‐rich polysaccharides extracted from abalone ( *Haliotis discus hannai* Ino). This suggests that certain extraction conditions may preferentially recover galactose‐dominant SP fractions, which could have implications for the functional and biological properties of the final product. In summary, our results demonstrate that the choice of extraction pH not only affects the overall yield of SPs but also significantly influences their monosaccharide composition. This highlights the importance of optimizing extraction parameters according to the desired structural and functional properties of the target polysaccharide.

### 3.6 Structural Properties of SPs Recovered From *O. mykiss* Head Using Acid and Alkaline Solubilization (pH Shift) Process

#### 3.6.1 FT‐IR Spectroscopy

**FIGURE 2:**
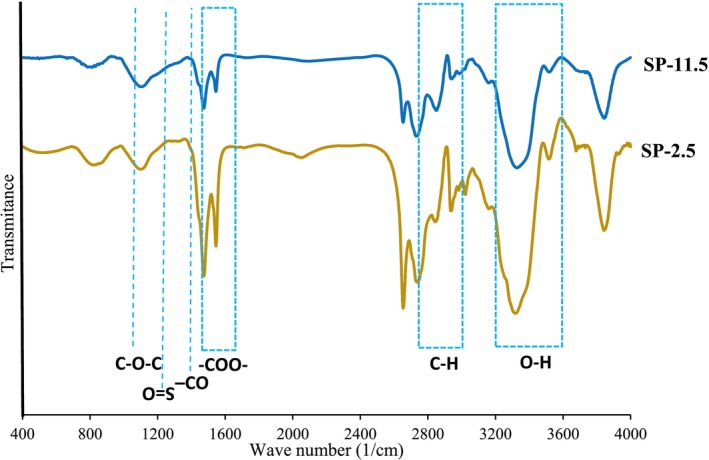
FTIR spectra of sulfated polysaccharides (SPs) recovered from *O. mykiss* head using the acid (SP‐2.5) and alkaline (SP‐11.5) versions of the pH shift method.

The FT‐IR spectra of the glycosaminoglycan samples, recorded in the range of 1400 to 4000 cm^−1^, are illustrated in Figure 2. While the overall spectral profiles of SP‐11.5 and SP‐2.5 were broadly similar, subtle differences were observed, which may be attributed to variations in the extraction source and the specific isolation procedures employed (Naghdi et al. 2023). These differences highlight the sensitivity of FT‐IR analysis to both the origin of the polysaccharide and the chemical environment during extraction. Both samples exhibited a broad absorption band between 3200 and 3600 cm^−1^, characteristic of O–H stretching vibrations, indicative of the presence of hydroxyl groups commonly found in polysaccharide structures. Additionally, the absorption observed in the 2700–3000 cm^−1^ region corresponds to C–H stretching vibrations, which are associated with methyl groups, such as those present in fucose residues (Olawuyi et al. 2020; Song et al. 2019). The presence of these functional groups confirms the polysaccharide nature of the extracted samples and suggests the retention of key structural features during the extraction process. The region between 1480 and 1640 cm^−1^ displayed distinct stretching bands attributed to the asymmetric stretching of carboxylate groups (–COO^−^), which are indicative of uronic acid residues within the sulfated polysaccharide chains (Jridi, Nasri, et al. 2019; Souissi et al. 2019). This observation is consistent with the chemical composition analysis, which revealed the presence of uronic acids in both SP‐11.5 and SP‐2.5, albeit at different concentrations. A prominent peak at 1245 cm^−1^ was observed, corresponding to the S=O stretching vibration of ester sulfate groups. Notably, this peak appeared more intense in the SP‐11.5 sample compared to SP‐2.5, aligning with the higher sulfate content determined for SP‐11.5 through chemical analysis. The increased intensity of this band suggests that alkaline extraction conditions may facilitate the recovery of SPs with greater degrees of sulfation, which could have important implications for their biological activity and functional properties (Jridi, Nasri, et al. 2019). Additional peaks detected at 1385 cm^−1^ and 1450 cm^−1^ are associated with O–C=O stretching and CO–CO stretching vibrations in carboxylic acid groups, respectively (Jridi, Nasri, et al. 2019). These bands further support the presence of uronic acid and other carboxyl‐containing monosaccharide residues within the extracted SPs. Overall, the FT‐IR spectra confirm the successful extraction of sulfated polysaccharides from rainbow trout heads using both acid and alkaline pH‐shift methods. The observed spectral features not only corroborate the chemical composition data but also provide insights into the structural integrity and functional group composition of the recovered SPs. Moreover, the more pronounced sulfate band in SP‐11.5 suggests that alkaline extraction may be preferable when targeting SPs with higher sulfation levels, which are often associated with enhanced bioactivity.

#### 3.6.2 Differential Scanning Calorimetry (DSC)

**FIGURE 3:**
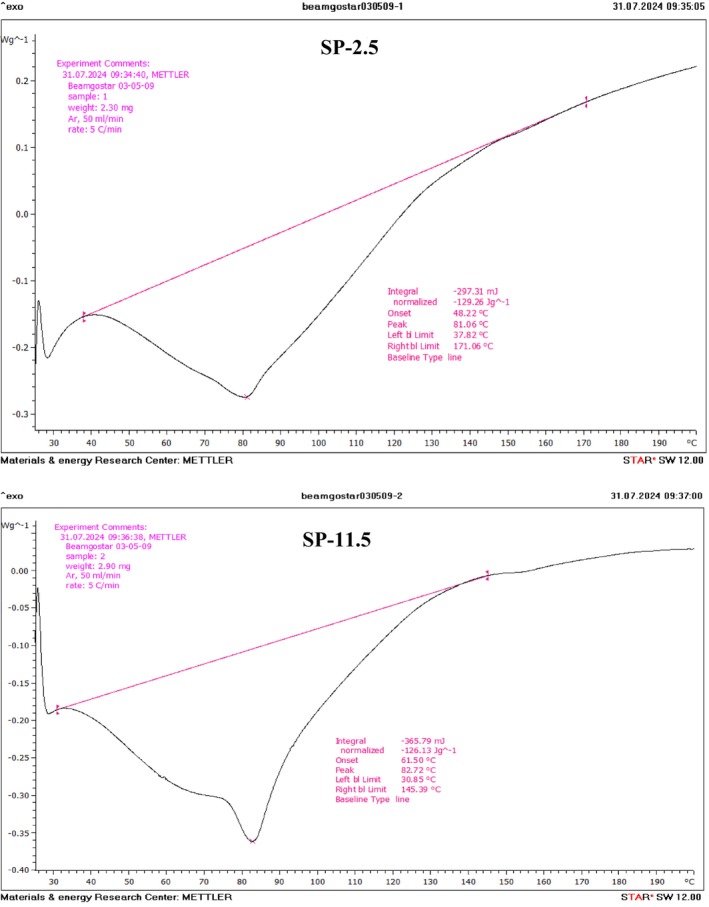
DSC thermograms of recovered GAGs from *O. mykiss* head using the acid (SP‐2.5) and alkaline (SP‐11.5) versions of the pH shift method.

The thermal stability of the recovered SPs from the acidic and alkaline solubilization process was assessed by employing differential calorimetric analysis (Figure 3). The thermal stability of the SPs is very important for their application in various fields; here, this analysis was conducted within a temperature range of 25°C to 200°C. The results show that the SP samples exhibit relatively similar thermal parameters, with a wide peak observed around 85°C. This peak is attributed to water loss from the sample and a change in the polymer structure (Krichen et al. 2018). It was also found that the onset temperature (To) and the conclusion temperature (Tc) for SP‐2.5 were 48.22°C and 171.06°C, respectively, while for SP‐11.5, they were 61.50°C and 145.39°C. Based on a previous study conducted by Zhu et al. (2017) it was established that a wide range between To and Tc for polysaccharide samples leads to low thermal stability. Also, the observed differences between the sample graphs may be related to variations in moisture content, the structure of the extracted sulfated polysaccharides, or the content of their hydrophilic groups (Krichen et al. 2018; Liu et al. 2013). Similar findings to the present study in previous studies conducted by Liu et al. (2013) and Naghdi et al. (2023) have made it clear that various extraction techniques can significantly affect the thermal stability of samples as revealed by DSC analysis.

#### 3.6.3 X‐Ray Diffraction (XRD) of Recovered SPs

**FIGURE 4:**
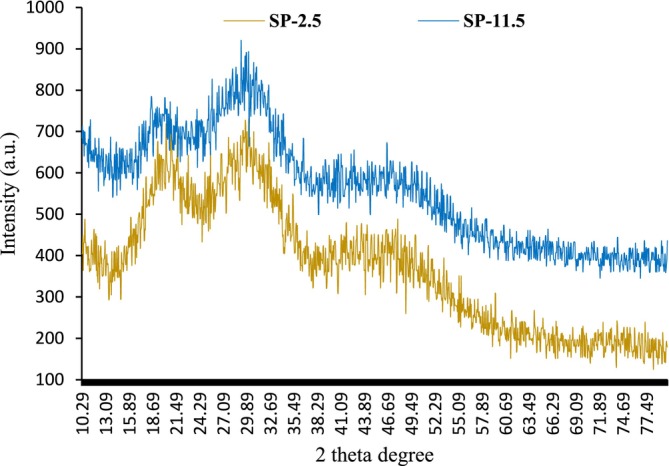
XRD graph of recovered sulfated polysaccharides (SPs) from *O. mykiss* head using the acid (SP‐2.5) and alkaline (SP‐11.5) versions of the pH shift method.

X‐ray diffraction (XRD) analysis was performed to evaluate the crystalline and amorphous characteristics of the recovered sulfated polysaccharide (SP) samples, as crystallinity plays a crucial role in determining various physicochemical and functional properties of biopolymers (Mokni Ghribi et al. 2015). The XRD patterns of both SP‐11.5 and SP‐2.5, recorded over a 2θ range of 5° to 80°, are shown in Figure 4. Both samples exhibited similar XRD profiles, characterized by two broad and diffuse peaks centered around 20° and 30° (2θ). The presence of these broad peaks, rather than sharp and well‐defined crystalline reflections, indicates that the amorphous phase predominates in the structure of the extracted SPs (Getachew et al. 2019; Nuerxiati et al. 2019). This amorphous nature is typical for many polysaccharides, which often lack long‐range molecular order due to their irregular and branched structures. However, the observed peaks also suggest a certain degree of short‐range order or semi‐crystallinity within the polysaccharide matrix. According to Ji, Wang, et al. (2022), such semi‐crystalline characteristics are commonly found in sulfated polysaccharides, where the coexistence of amorphous and crystalline domains can arise from the heterogeneous arrangement of monosaccharide units and the presence of sulfate and uronic acid groups. Understanding the crystallinity of SPs is particularly important, as it directly influences key material properties such as tensile strength, flexibility, solubility, swelling behavior, and dispersion in aqueous systems (Getachew et al. 2019; Saravana et al. 2016). For instance, higher amorphous content generally enhances solubility and swelling capacity, which could be advantageous for certain food, pharmaceutical, or biomedical applications. Conversely, a higher degree of crystallinity may impart greater mechanical strength but lower solubility. In this study, the similar XRD patterns observed for both SP‐11.5 and SP‐2.5 suggest that the pH‐shift extraction process, whether conducted under alkaline or acidic conditions, does not markedly alter the overall crystalline structure of the recovered SPs. This finding is consistent with the FT‐IR and DSC results, which also indicated preservation of the main structural features regardless of extraction pH. The predominance of the amorphous phase in both samples may also contribute to their observed functional properties, such as enhanced water solubility and potential bioactivity. In summary, XRD analysis confirmed that the SPs extracted from rainbow trout heads by both acid and alkaline pH‐shift methods are predominantly amorphous with some semi‐crystalline features. This structural characteristic is likely to influence their application potential in various biorefinery and value‐added product contexts.

### 3.7 Antioxidant Activity of Recovered SP Samples From *O. mykiss* Head Using Acid and Alkaline Solubilization (pH Shift) Process

#### 3.7.1 DPPH Radical Scavenging Assay

**FIGURE 5:**
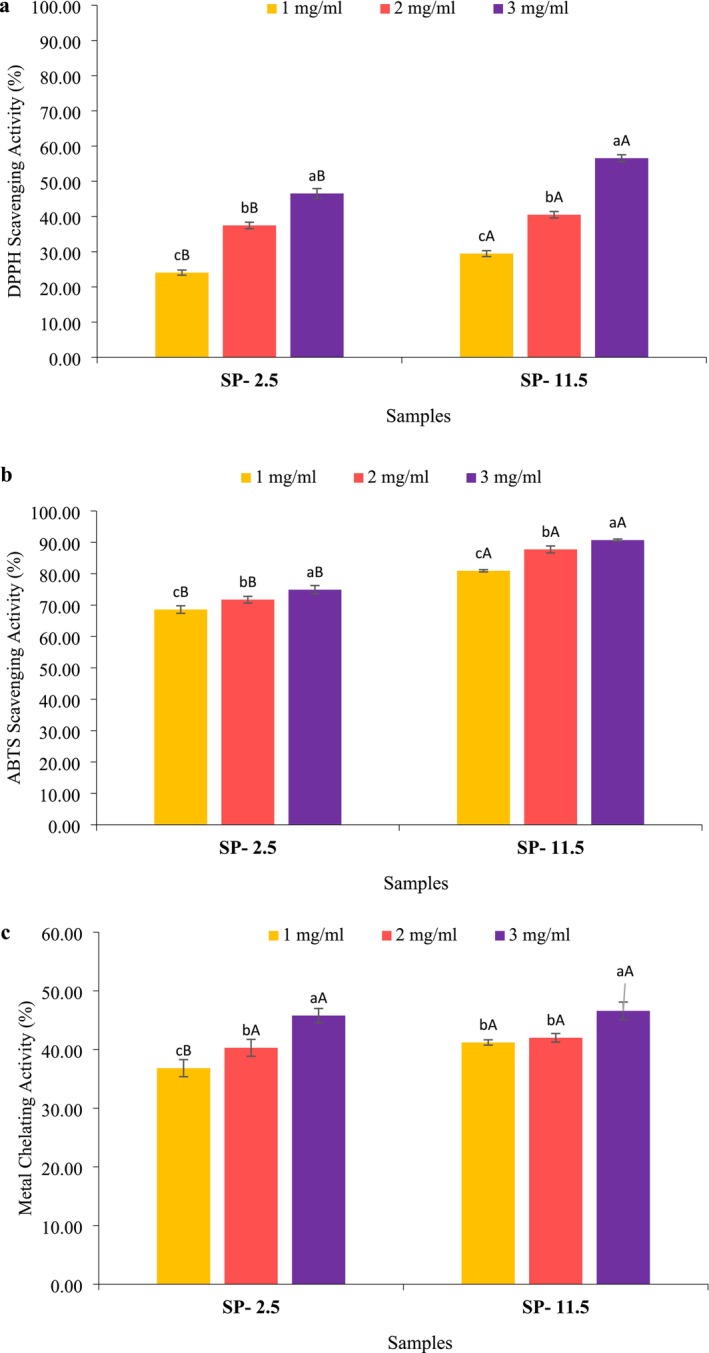
DPPH radical scavenging activity (a), ABTS radical scavenging (b), and iron (Fe^2+^) chelating activity (c) of sulfated polysaccharides (SP) recovered from *O. mykiss* by‐products using the acid (SP‐2.5) and alkaline (SP‐11.5) versions of the pH shift method. The capital letters show the difference between various samples in similar concentrations. The small letters show the difference between various concentrations of the one sample.

The DPPH radical scavenging activity of the extracted SPs is shown in Figure 5a. The results represented a positive correlation between the SP concentrations and their antioxidant activity against DPPH radical (*p* < 0.05). This trend aligns with previous studies, suggesting that this phenomenon could be related to an increase in radical‐scavenging components within the sample (Jia et al. 2020). Furthermore, SP‐11.5 consistently exhibited significantly higher DPPH radical scavenging activity compared to SP‐2.5 at all concentrations tested. These results can be attributed to the higher sulfate and carbohydrate content of SP‐11.5 (Krichen et al. 2016). Bioactive compounds like polysaccharides, proteins, and lipids can exhibit their antioxidant activity by donating electrons to the DPPH free radical, functioning as hydrogen donors (Jridi, Abdelhedi, et al. 2019; Souissi et al. 2019). In this study, at a concentration of 1 mg/mL, SP‐11.5 and SP‐2.5 exhibited 29.46% and 24.05% DPPH radical scavenging activity, respectively. These results are comparable to those reported by Souissi et al. (2019) for evaluating the antioxidant properties of the extracted sulfated polysaccharides from the *Solen marginatus* at the same concentration. Similarly, the antioxidant properties of sulfated polysaccharides from squid skin and muscle documented by Jridi, Abdelhedi, et al. (2019) align closely with the findings of the present study.

#### 3.7.2 ABTS Radical Scavenging Activity

The results of the antioxidant properties of the SP samples obtained in the ABTS free radical activity test are shown in Figure 5b. The results showed that there is a significant difference between the antioxidant properties of all the investigated concentrations of the extracted samples. Also, in similar concentrations between the samples, SP‐11.5 significantly showed higher scavenging properties (*p* < 0.05). These results align with the previous section's finding on DPPH free radical scavenging of the recovered SPs. It has been previously determined that the ABTS radical chelating activity of polysaccharides can be linked to various factors such as molecular weight (Li and Wang 2016) and sulfate amount (Khan et al. 2019). In this regard, it has been pointed out that extraction methods significantly influence the ABTS radical chelating properties of the samples due to variations in polysaccharide composition and purity (Grina et al. 2020). In the present study, the sulfate content of SP‐11.5 samples was higher than that of SP‐2.5, and the same trend was also obtained for protein content, which confirmed the obtained results. It has even been reported that sample hydroxyl content affects ABTS free radical scavenging, as hydroxyl reduction during drying reduces scavenging activity (Hu et al. 2017). A study conducted by Cho et al. (2019) on the antioxidant evaluation of polysaccharides extracted from African snails found that the highest antioxidant activity was at a concentration of 10 mg/mL with a value of 30.32%. This value was lower than our achieved results at a concentration of 1 mg/mL.

#### 3.7.3 Iron (Fe^2+^) Chelating Activity

Iron (Fe^2+^), essential as the active center in many oxidation reaction catalysts, typically reacts with oxygen (O_2_) in the body and produces harmful superoxide and hydroxyl radicals (Ji, Liu, et al. 2022). However, chelating Fe^2+^ by antioxidant compounds decreases the production of these reactive oxygen species, thereby indirectly showing the antioxidant activity of the bioactive compound such as sulfated polysaccharide (Ji, Liu, et al. 2022). The metal ion chelating activity of the recovered SP samples (Figure 5c) showed that SP‐11.5 exhibited significantly higher chelating activity compared to the other samples (*p* < 0.05). The highest chelating properties of SP‐11.5 and SP‐2.5 were 51.92% and 45.79%, respectively, observed at a concentration of 3 mg/mL. Studies on the antioxidant properties of sulfated polysaccharides from skin and meat have shown that sulfate groups play a crucial role in their reducing power (Jridi, Nasri, et al. 2019). Notably, sample SP‐11.5 displayed a significantly higher sulfate content compared to the other samples, potentially contributing to its enhanced reductive activity.

### 3.8 Antibacterial Properties

**FIGURE 6:**
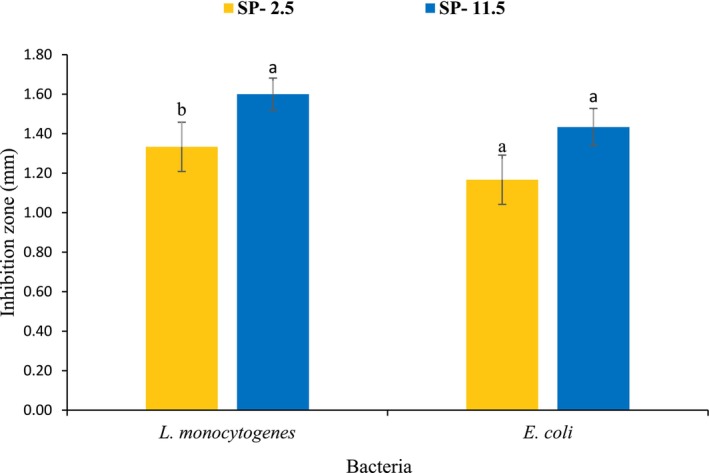
Antibacterial activity of sulfated polysaccharides (SPs) recovered from *O. mykiss* by‐products using the acid (SP‐2.5) and alkaline (SP‐11.5) versions of the pH shift method.

Figure 6 presents the results of the antibacterial properties of the recovered sulfated polysaccharides. The results displayed that SP‐11.5 exhibited significantly better antibacterial activity against *L. monocytogenes* and *E. coli* compared to SP‐2.5. The inhibition halo against *L. monocytogenes* was 1.6 mm for SP‐11.5 and 1.33 mm for SP‐2.5 (*p* < 0.05), while for *E. coli* bacteria, the inhibition zone was 1.43 mm and 1.17 mm, respectively (*p* > 0.05). As can be seen from the result, *E. coli* demonstrated a higher resistance to sulfated polysaccharides than *L. monocytogenes* , supporting the statements made by Jridi, Abdelhedi, et al. (2019) and Shanmugam et al. (2008). This is due to the distinctive structure of Gram‐negative bacteria compared to Gram‐positive bacteria (Zeinab et al. 2023). Gram‐negative bacteria, such as *E. coli* , possess a unique outer layer composed of lipopolysaccharide (LPS), which is absent in gram‐positive bacteria (Silhavy et al. 2010). This LPS layer serves as a protective effect against antimicrobial agents and disinfectants. Additionally, these bacteria have a thinner peptidoglycan layer, providing reduced resistance to the entry of antimicrobial agents, consequently leading to increased resistance (Silhavy et al. 2010; Zeinab et al. 2023). Our antibacterial results for SP samples at a concentration of 1 mg/mL were much lower than those documented by Jridi, Abdelhedi, et al. (2019), who used considerably higher concentrations (25 and 50 mg/mL) of sulfated polysaccharides. Furthermore, the dose‐dependent antibacterial activity of fish‐waste sulfated polysaccharides reported by Naghdi et al. (2023) suggested that sample concentration can significantly affect the antibacterial activity of SPs, which confirms the difference between our results and Jridi's reported results (Jridi, Abdelhedi, et al. 2019). Notably, Krichen et al. (2015) declared that sulfated polysaccharides extracted from shark skin have no antibacterial properties against *E. coli* at higher concentrations of 50 and 100 mg/mL. Abdelhedi et al. (2016) revealed that precipitation of sulfated polysaccharides using ethanol and cetylpyridinium chloride (CPC) significantly affected the antibacterial activity of sulfated polysaccharides against a range of gram‐positive and gram‐negative bacteria. Souissi et al. (2019) declared that deproteinization of sulfated polysaccharides enhanced their antibacterial activity against Gram‐negative bacteria but diminished it against Gram‐positive bacteria. Interestingly, our SP‐11.5 sample, which had a higher protein content than SP‐2.5, presented the best antibacterial properties, indicating that the sample protein content can play a complex function in the antibacterial activity of sulfated polysaccharides. Additionally, Li and Shah (2014) revealed that sulfation of polysaccharides could improve the antibacterial effects of polysaccharides by disrupting bacterial cell walls. In line with these findings, our study recommends that the higher sulfate content of the SP‐11.5 sample may contribute to its superior antibacterial activity compared to the SP‐2.5 sample.

## 4 Conclusions

The classic pH‐shift method was successfully extended for sequential extraction of functional proteins and sulfated polysaccharides from the filleting by‐products of rainbow trout. The results indicated that the yield of protein isolate, as well as its solubility and intrinsic fluorescence, was higher in the IP‐11.5 sample compared to IP‐2.5, while the content of active sulfhydryl groups showed the opposite trend. Furthermore, the functional properties assessed in this study were consistently superior in IP‐11.5 across all tests. The extraction yield of sulfated polysaccharides using the alkaline method (SP‐11.5) was significantly higher than that obtained with the acidic pH method (SP‐2.5), almost doubling the yield. Chemical composition analysis of the extracted sulfated polysaccharides showed that the carbohydrate and sulfate content was significantly higher in SP‐11.5 than in SP‐2.5, while uronic acid content was higher in the acidic sample. FTIR, XRD, and DSC analyses revealed no significant differences between the SP samples, which were found to consist of a mixture of rhamnose, mannose, glucose, and galactose monosaccharides. Notably, SP‐11.5 exhibited superior antioxidant properties in DPPH, ABTS, and metal ion chelation tests compared to SP‐2.5. Additionally, this sample demonstrated higher antibacterial activity against *L. monocytogenes* and *E. coli* when compared to SP‐2.5. Overall, based on these results, we conclude that the pH‐shift process presents an innovative and promising approach for the sequential extraction of sulfated polysaccharides from aquatic byproducts. A biorefinery platform, this method could serve as a viable alternative to enzymatic hydrolysis for sulfated polysaccharide extraction, but further research will be necessary to fully realize this potential.

## Author Contributions

**Shahab Naghdi:** conceptualization, investigation, methodology, formal analysis, writing – original draft, writing – review and editing. **Masoud Rezaei:** supervision, project administration, conceptualization, resources, writing – review and editing. **Mehdi Tabarsa:** investigation, formal analysis. **Mehdi Abdollahi:** conceptualization, writing – review and editing.

## Conflicts of Interest

The authors declare no conflicts of interest.

## Data Availability

The data that support the findings of this study are available from the corresponding author upon reasonable request.
